# Frailty transition and depression among community-dwelling older adults: the Korean Longitudinal Study of Aging (2006–2020)

**DOI:** 10.1186/s12877-022-03570-x

**Published:** 2023-03-17

**Authors:** Nataliya Nerobkova, Yu Shin Park, Eun-Cheol Park, Jaeyong Shin

**Affiliations:** 1 Department of Public Health, Graduate School, Yonsei University, Seoul, Republic of Korea; 2 Institute of Health Services Research, Yonsei University, 50 Yonsei-to, Seodaemun-Gu, Seoul, 03722 Republic of Korea; 3 Department of Preventive Medicine, Yonsei University College of Medicine, 50 Yonsei-to, Seodaemun-Gu, Seoul, 03722 Republic of Korea; 4 Department of Policy Analysis and Management, College of Human Ecology, Cornell University, Ithaca, NY USA

**Keywords:** Frailty, Depression, CES-D-10, Community-dwelling population, Longitudinal study

## Abstract

**Background:**

Frailty is recognized as a geriatric syndrome associated with depression. The consequences and mechanism of frailty transitions are still understudied. This study assessed the influence of frailty transitions on new-onset depressive symptomology using longitudinal, nationwide data of Korean community-dwelling older adults.

**Methods:**

Longitudinal population-based study conducted in every even-numbered year starting from 2006 to 2020 (eight waves) with a sample of older adults aged ≥ 60 years old. After the application of exclusion criteria, a total of 2,256 participants were included in the 2008 baseline year. Frailty transition was determined through the biennial assessment of change in frailty status using the frailty instrument (FI); depression was measured using the Center for Epidemiological Studies Depression 10 Scale. We employed the lagged general estimating equations to assess the temporal effect of frailty transition on obtaining depressive symptoms.

**Results:**

Compared to non-frail individuals, the risk of depression was higher in transitioned into frailty and constantly frail participants over a 2-year interval: men (odds ratio (OR) 1.26, 95% confidence interval (CI) 1.21–1.32; OR 1.29, 95% CI 1.21–1.38), women (OR 1.34, 95% CI 1.28–1.40; OR 1.51, 95% CI 1.41–1.62), respectively.

**Conclusions:**

Frailty transition is found to be associated with new-onset depressive symptoms. Frail individuals and those who transitioned into frailty were associated with a higher risk of depression. Particular attention should be paid to these frailty transitioned groups. Early intervention and implementation of prevention strategies at physical, nutritional, and social levels are warranted to ameliorate frailty and depression in late life.

**Supplementary Information:**

The online version contains supplementary material available at 10.1186/s12877-022-03570-x.

## Background

Frailty is an aging-related condition highly prevalent in the older population and emerging as a risk factor for adverse health outcomes, including falls, disability, hospitalization, and an increased risk of morbidity and mortality [1–4]. As frailty is a geriatric syndrome that severely affects the aging population, it has gained increasing attention among researchers.

In South Korea (hereafter, Korea), an aging population and a decline in birth rate are the greatest public health concerns [4–6]. The proportion of the general aged population is expected to increase substantially to 24.5% by 2030 and 41.0% by 2060 [7]. Thus, measures for curbing the incidence of frailty among older adults are warranted.

Frailty syndrome is a broad concept with various causative risk factors. Numerous instruments [8, 9] and scales have been developed to measure frailty syndrome [10, 11]. However, some factors, including weakening of handgrip strength and self-reported exhaustion, are common issues in the use of models [8]. At present, frailty is considered a multidimensional dynamic measure based on various age-related deficits [12, 13], as opposed to the earlier perception of frailty in a non-dimensional and only physical manner [14, 15]. Frailty is a dynamic condition, and its changes are characterized by a transition to a worsened or improved state over time. The frailty instrument (FI), a frailty measure that was developed and validated for the Korean population, is utilized for rapid assessment of frailty and determination of adverse health outcomes in older adults [4, 16, 17]. The FI is based on a broader approach to the measurement of frailty and includes physical (handgrip strength), psychological (exhaustion), and social (social isolation) factors. Evaluating the changes in frailty over time using the FI allows for consideration of the bidirectional aspect of transitions in frailty status.

The dynamic nature of frailty has been investigated in some longitudinal studies. However, most of these previous studies focused on the predictive risk factors of frailty transitions rather than on frailty transition outcomes as a changing continuous risk factor itself. Previous longitudinal studies conducted in Korea have established the impact of frailty transition on the cognitive functions of older adults [4, 18]. However, the impact of frailty transition on depressive symptoms among older Korean adults remains unclear.

Depression is a well-known risk factor for many health-related conditions [19–21]. Hence, studies have been conducted with the aim of preventing, slowing, and ameliorating depressive symptoms in vulnerable populations. The association between frailty and depression has been evaluated in several cross-sectional and longitudinal studies. However, little attention has been paid to the relationship between changes in frailty status over time and the development of depressive symptoms. Hypotheses of comparable biological mechanisms of frailty and depression have been proposed [22]. Although the results of cross-sectional studies indicate a positive association between depression and frailty [23, 24], findings from cohort studies are less consistent [25]. In addition, several studies conducted to examine the bidirectional relationship between depression and frailty showed controversial results [26–28].

To date, little is known about the effect of more comprehensive conceptualizations of frailty and its transitions on the development of depressive symptoms. Therefore, the aim of this study was to investigate the effect of frailty transitions on new-onset depressive symptoms among community-dwelling older adults in Korea using the FI and the Center for Epidemiological Studies Depression 10 Scale (CES-D-10).

## Methods

### Data source and sample

**Fig. 1:**
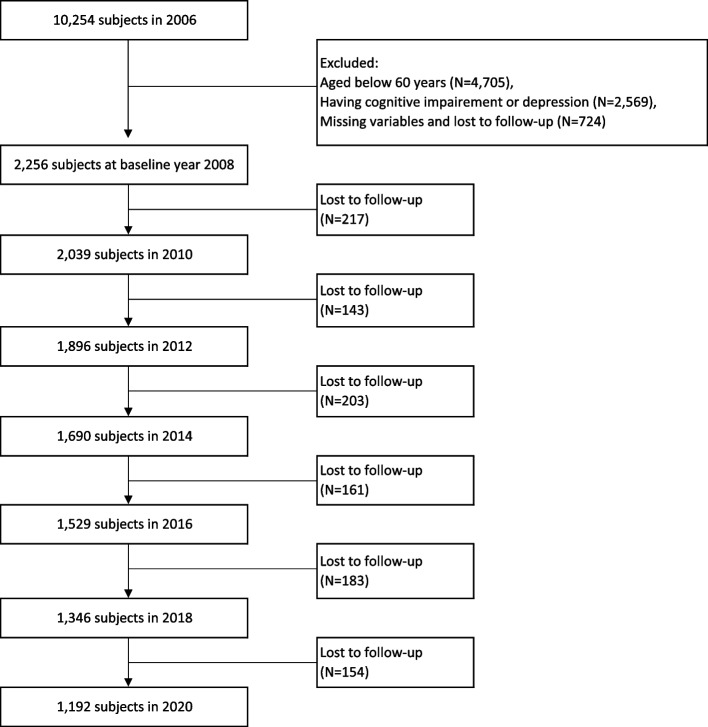
Flowchart of the study participants from 2006 to 2020

This study was conducted using data collated over 12 years from the first to the eighth wave (2006 to 2020) of the Korean Longitudinal Study of Aging. Since its establishment in 2006, the Korea Labor Institute has been collecting regular panel data of the same population sample of older adults aged more than 45 years from all regions in Korea. The total number of participants surveyed in 2008 was 8,688 (approximately 84.7% of the original 10,254 participants surveyed in 2006). The survey was conducted every even-numbered year starting from 2006, primarily using the same survey categories. The sample retention rate in 2020 was 63.3%. Information on the family background, demographic characteristics, family composition, health, employment, income, assets, and subjective quality of life of the respondents were collected for the survey [29]. Additional information about the survey is available on the panel survey organization website (https://survey.keis.or.kr/klosa/klosa01.jsp). The exclusion criteria for the survey included cognitive impairment and depression status during the first wave (2006), age below 60 years, missing information on the employed variables, and loss to follow-up. Application of these criteria led to the inclusion of 2,256 participants in 2008, 2,039 in 2010, 1,896 in 2012, 1,690 in 2014, 1,529 in 2016, 1,346 in 2018, and 1,192 in 2020. The selection process of the participants is shown in detail in Fig. 1.

The KLoSA survey was approved by the National Statistical Office and Institutional Review Board of the Korea Centers for Disease Control and Prevention. All methods were conducted in accordance with the relevant guidelines and regulations. As the KLoSA database has been published to the public for scientific use, ethical approval was not required for the study. All participants were required to provide written informed consent to participate in the KLoSA survey and agreed to be used in further scientific research. The data were anonymized and de-recognizable with no personal information, with cautious protection on confidentiality.

### Variables

The variable of interest, “frailty transition,” was assessed as a time-varying covariate that reflects changes in frailty status as defined using the FI, which was developed and validated using the community-dwelling older adult population of Korea. The FI allows for rapid assessment of frailty and associated adverse outcomes, including disability, morbidity, institutionalization, and mortality, and has high predictive validity, discrimination, and calibration power [29]. The FI depicts the sociopsychological and physical components of frailty based on three criteria: exhaustion, social isolation, and weakness of handgrip strength [4, 17, 30]. The exhaustion criterion is estimated using self-reported measures of feeling that every task required effort during the previous week. Social isolation status is determined if respondents report not participating in any social group activity. Handgrip weakness is evaluated using sex-specific grip strength thresholds: < 24 kg for men and < 15 kg for women. The three variables are graded using a three-point scale, with ≥ 2 points classified as frail and ≤ 1 point as non-frail. In the survey, the lag function was used to detect changes in frailty status in the prior and the succeeding waves, following a two-year gap. Therefore, frailty transitions were categorized into four groups: (1) Non-frail → Non-frail, (2) Non-frail → Frail, (3) Frail → Frail, and (4) Frail → Non-frail.

The outcome variable, “depression,” was identified by measuring depressive symptoms using the CES-D-10. The 10-item version of the CES-D, established on the work of Andresen et al., was extrapolated from the original 20-item version of the CES-D by applying item-total correlations and eliminating redundant items [31]. The CES-D-10 is a validated screening tool used to identify major depressive symptoms in older adults [32–34]. The validity of the Korean version of CES-D-10 for screening of depressive symptoms is well based [35, 36]. Responses are graded on a four-point scale, coded 0–1, with a total score of 10 points. Higher scores indicate greater distress. A cut-off score of ≥ 4 points was set for the detection of depression in the survey participants, which is consistent with the proposed use of the CES-D-10 as a screening instrument [31, 37, 38].

Data on sociodemographic characteristics and health-related conditions were added as potential confounders in this study. Sociodemographic characteristics included sex (men, women), age (60–69, 70–79, ≥ 80 years), educational level (middle school or below, high school or above), marital status (married, not married), occupational status (working, not working) and income level per month in quartiles (low, middle-low, middle-high, and high). Additionally, we considered the participants’ regions of residence (urban or rural areas). Limitations in activities of daily living (ADL) were determined if the respondents had difficulty performing any daily, necessary tasks, including getting dressed, washing their face and hands, bathing, eating meals, leaving a room, and using the toilet. Limitations in Instrumental Activities of Daily Living (IADL) were defined as difficulties with performing social function-related tasks, including making/receiving phone calls, managing finances, companionship, mental support, transportation usage, household chores, preparation of meals, shopping, taking medications, and doing laundry. Cognitive function was assessed using the Korean version of the Mini-Mental State Examination (MMSE). The MMSE is a 30-point questionnaire, with 24 points being the cut-off for cognitive impairment. The chronic diseases considered in the present study included hypertension, diabetes mellitus, cancer, lung disease, heart disease, and cerebrovascular disease. Comorbidities were grouped into three categories depending on the number of diseases a participant had (0, 1, or ≥ 2 diseases). In addition, we considered smoking status (smoker, non-smoker), body mass index (normal, abnormal: underweight and overweight), and life satisfaction (bad, normal, and good).

### Statistical analysis

We evaluated relationships between the two-year frailty transition and CES-D-10 score using a 2-year lagged multivariable lagged generalized estimating equations (GEE) model that is an extension of the quasi-likelihood approach used to analyze longitudinal correlated data. The GEE model allows for repeated measurement analysis of longitudinal panel survey data and considers the correlation within the subject to generate odds ratios (ORs) and 95% confidence intervals (CIs), and the corresponding p-value. All statistical analyses were performed separately for men and women to examine sex-specific differences in terms of the diverse impact of frailty transition on depressive symptoms. A total of eight waves were used for the analysis, and repeated measurements were carried out for each individual up to seven times. Two-year lagged changes in frailty transition were calculated using the frailty status in the preceding and follow-up waves (2006–2008, 2008–2010, 2010–2012, 2012–2014, 2014–2016, 2016–2018, and 2018–2020) following a two-year interval. Furthermore, a subgroup analysis was performed to reveal the relationship between frailty transition and depression status. We estimated the lagged GEE analyses for each FI with respect to the CES-D-10 score. Differences were considered statistically significant with a *p*-value of < 0.05. Statistical analyses were performed using the GENMOD procedure in SAS (version 9.4; SAS Institute Inc., Cary, NC, USA) with *link identity* and *distribution normal*.

## Results

The sex-stratified baseline characteristics of the study population are summarized in Table 1. A total of 2,256 people were included in the survey in the baseline year (1,256 men and 1,000 women). The percentage of women with a CES-D-10 score ≥ 4 was almost twice that of men (14.6 and 8.8%, respectively). Regarding frailty status, 39.3% of the men and 59.1% of the women transitioned into frailty, and 45% of the men and 44% of the women with a sustained frailty status showed depressive symptoms. There were significant differences in other covariates, such as age, occupational status, ADL, IADL, and MMSE status, between men and women with a CES-D-10 score ≥ 4. CES-D-10 score distributions for the main variables were additionally summarized as the median and interquartile range (Supplementary Table 1).

**Table 1:** General characteristics of the study population (baseline 2006→2008)

Variables	Center of Epidemiologic Studies Depression Scale, 10-item version (CES-D-10)											
	**Men**						**Women**					
	**Total**		**< 4**		**≥ 4**		**Total**		**< 4**		**≥ 4**	
	**N**	**%**	**N**	**%**	**N**	**%**	**N**	**%**	**N**	**%**	**N**	**%**
**Total *N* = 2256**	1256	100.0	1146	91.2	110	8.8	1000	100.0	854	85.4	146	14.6
**Frailty status**												
Non-frail → Non-frail	1112	88.5	1051	94.5	61	5.5	847	84.7	765	90.3	82	9.7
Non-frail → Frail	84	6.7	51	60.7	33	39.3	88	8.8	36	40.9	52	59.1
Frail → Frail	20	1.6	11	55.0	9	45.0	25	2.5	14	56.0	11	44.0
Frail → Non-frail	40	3.2	33	82.5	7	17.5	40	4.0	39	97.5	1	2.5
**Age**												
60–69	661	52.6	620	93.8	41	6.2	576	57.6	513	89.1	63	10.9
70–79	497	39.6	447	89.9	50	10.1	373	37.3	305	81.8	68	18.2
≥ 80	98	7.8	79	80.6	19	19.4	51	5.1	36	70.6	15	29.4
**Region**												
Urban area	543	43.2	504	92.8	39	7.2	471	47.1	408	86.6	63	13.4
Rural area	713	56.8	642	90.0	71	10.0	529	52.9	446	84.3	83	15.7
**Educational level**												
Middle school or below	490	39.0	436	89.0	54	11.0	674	67.4	566	84.0	108	16.0
High school or above	766	61.0	710	92.7	56	7.3	326	32.6	288	88.3	38	11.7
**Occupational status**												
Working	529	42.1	499	94.3	30	5.7	174	17.4	157	90.2	17	9.8
Non-working	727	57.9	647	89.0	80	11.0	826	82.6	697	84.4	129	15.6
**Marital status**												
Married	1164	92.7	1068	91.8	96	8.2	644	64.4	558	86.6	86	13.4
Not married	92	7.3	78	84.8	14	15.2	356	35.6	296	83.1	60	16.9
**Household income**												
Quartile 1 (low)	472	37.6	409	86.7	63	13.3	440	44.0	370	84.1	70	15.9
Quartile 2	379	30.2	355	93.7	24	6.3	276	27.6	250	90.6	26	9.4
Quartile 3	240	19.1	227	94.6	13	5.4	159	15.9	130	81.8	29	18.2
Quartile 4 (high)	165	13.1	155	93.9	10	6.1	125	12.5	104	83.2	21	16.8
**Chronic disease**												
0	593	47.2	545	91.9	48	8.1	439	43.9	393	89.5	46	10.5
1	447	35.6	404	90.4	43	9.6	378	37.8	311	82.3	67	17.7
2 or more	216	17.2	197	91.2	19	8.8	183	18.3	150	82.0	33	18.0
**ADL**												
Normal	1239	98.6	1138	91.8	101	8.2	989	98.9	849	85.8	140	14.2
Abnormal	17	1.4	8	47.1	9	52.9	11	1.1	5	45.5	6	54.5
**IADL**												
Normal	1103	87.8	1020	92.5	83	7.5	950	95.0	818	86.1	132	13.9
Abnormal	153	12.2	126	82.4	27	17.6	50	5.0	36	72.0	14	28.0
**MMSE**												
≥ 24	1088	86.6	1017	93.5	71	6.5	751	75.1	671	89.3	80	10.7
< 24	168	13.4	129	76.8	39	23.2	249	24.9	183	73.5	66	26.5
**Smoking status**												
Non-smoker	491	39.1	455	92.7	36	7.3	977	97.7	838	85.8	139	14.2
Smoker	765	60.9	691	90.3	74	9.7	23	2.3	16	69.6	7	30.4
**BMI**												
Normal	1188	94.6	1087	91.5	101	8.5	932	93.2	806	86.5	126	13.5
Abnormal	68	5.4	59	86.8	9	13.2	68	6.8	48	70.6	20	29.4
**Satisfaction of Life**												
Bad	161	12.8	132	82.0	29	18.0	150	15.0	101	67.3	49	32.7
Normal	777	61.9	709	91.2	68	8.8	602	60.2	520	86.4	82	13.6
Good	318	25.3	305	95.9	13	4.1	248	24.8	233	94.0	15	6.0

Table 2 depicts the findings of the lagged GEE model analyses of the association between changes in frailty status and the risk for a CES-D-10 score ≥ 4. We noted that in both men and women, those who showed a Non-frail → Frail transition (men: OR 1.26, 95% CI 1.21–1.32; women: OR 1.34, 95% CI 1.28–1.40) and Frail → Frail transition (men: OR 1.29, 95% CI 1.21–1.38; women: OR 1.51, 95% CI 1.41–1.62) had higher ORs than non-frail older adults. Owing to a large number of missing data and participants lost to follow-up, as well as the overlap of the exhaustion item with CES-D-10, several sensitivity analyses (Supplement Tables 2, 3, 4) were performed. The received findings were mainly consistent with the primary outcome.

**Table 2:** Generalized linear model using the GEE with CES-D-10 score in 2008–2020

Variables	CES-D-10 score ≥			
	**Men**		**Women**	
	**OR**^**a**^	**95% CI**	**OR**^**a**^	**95% CI**
**Frailty status**				
Non-frail → Non-frail	1.00		1.00	
Non-frail → Frail	1.26	(1.21—1.32)	1.34	(1.28—1.40)
Frail → Frail	1.29	(1.21—1.38)	1.51	(1.41—1.62)
Frail → Non-frail	1.04	(1.00—1.08)	1.00	(0.96—1.04)

^a^ Adjusted for other covariates

**Fig. 2:**
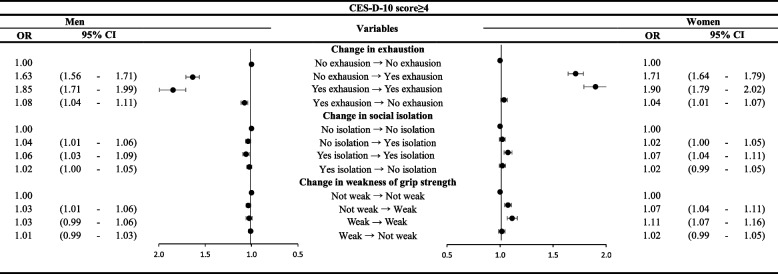
Subgroup analysis of Frailty Instrument (FI) components with depression. The exhaustion domain of the FI showed the most significant association with depression

Figure 2 shows the lagged GEE model analysis results of the effect of the two-year changes in FI status on the risk of depressive symptoms. We observed statistically significant associations between depressive symptoms and each change in FI status. However, the most significant association was between depressive symptoms and the exhaustion domain of the FI. Men and women who transitioned into an exhausted state (men: OR 1.63, 95% CI 1.56–1.71; women: OR 1.71, 95% CI 1.64–1.79) or maintained an exhausted state (men: OR 1.85, 95% CI 1.71–1.99; women: OR 1.90, 95% CI 1.79–2.07) had higher ORs than their non-exhausted counterparts.

The findings of the independent subgroup analysis of the variables associated with the effect of changes in frailty status on a CES-D-10 score ≥ 4 are shown in Table 3. The results indicated that the Non-frail → Frail and the Frail → Frail groups had the highest ORs among participants who were experiencing cognitive impairment: MMSE score lower than 24 points was significantly associated with depressive symptoms: Non-frail → Frail (men: OR 1.25, 95% CI 1.17–1.33; women OR 1.39, 95% CI 1.31–1.48), Frail → Frail (men: OR 1.36, 95% CI 1.23–1.50; women OR 1.62, 95% CI 1.49–1.76).

**Table 3:** Subgroup analysis using the GEE of CES-D-10 score with frailty transition in 2006–2020

Variables	CES-D-10 score ≥ 4													
	**Men**							**Women**						
	**Non-frail → Non-frail**	**Non-frail → Frail**		**Frail → Frail**		**Frail → Non-frail**		**Non-frail → Non-frail**	**Non-frail → Frail**		**Frail → Frail**		**Frail → Non-frail**	
	**OR**	**OR**^**a**^	**95% CI**	**OR**^**a**^	**95% CI**	**OR**^**a**^	**95% CI**	**OR**	**OR**^**a**^	**95% CI**	**OR**^**a**^	**95% CI**	**OR**^**a**^	**95% CI**
**MMSE**														
≥ 24	1.00	1.29	(1.22—1.35)	1.25	(1.15—1.36)	1.04	(0.99—1.08)	1.00	1.29	(1.21—1.38)	1.40	(1.26—1.55)	1.03	(0.99—1.08)
< 24	1.00	1.25	(1.17—1.33)	1.36	(1.23—1.50)	1.06	(0.99—1.14)	1.00	1.39	(1.31—1.48)	1.62	(1.49—1.76)	0.98	(0.93—1.04)

^a^ Adjusted for other covariates

## Discussion

Depression is a common medical illness among older adults that is associated with numerous adverse health outcomes. The potential risk factors for the development of late-life depression likely comprise complex interactions among genetic factors, cognitive dysfunction, age-associated neurobiological fluctuations, and stressful events [39]. Thus, strategies developed through a detailed and precise examination of the above-mentioned risk factors and specifically designed to minimize the risks of depression and maintain well-being in later life are warranted. In the present study, we investigated the association between frailty transition and the onset of depressive symptoms among community-dwelling Korean adults over 60 years old. The results showed that frailty (transition into frailty or maintenance of frailty over a two-year period) was significantly associated with new-onset depressive symptomatology compared with continuous non-frailty. Furthermore, we suggest that transitional endpoints, particularly transitioning to a frailty state, might be the main features correlated with depression, given that baseline status may only influence the effects on follow-up status. Notably, the results also indicated that while improvement of frailty in men reduced depressive symptoms, participants still showed signs of depression compared to their non-frail counterparts.

The relationships between older age, frailty, and depression have been evaluated in previous studies. The results of the studies demonstrated a bidirectional association between frailty and depression. In addition, several prospective studies have been conducted to examine whether the presence or absence of frailty at baseline predicts new-onset incident depression. In a population-based cohort study of older adults aged ≥ 65 years who were followed up at 3, 6, and 9 years, 30.6% of the participants without depression developed a depressed mood during follow-up, and the frail state was associated with a significant risk of new onset of depression in adjusted models [40]. In another study, follow-up analysis at 2 and 4 years revealed significant associations between frailty and the onset of depression in adjusted models [41]. These findings and those of the present study suggest that frailty status and transition are key causes of emotional distress (such as feelings of worthlessness or hopelessness) [42], which, in turn, may result in new-onset depressive symptomatology.

In the present study, subgroup analysis of independent variables indicated that respondents with cognitive impairment during follow-up showed an association between frailty status or transition to frailty and new onset of depressive symptoms. Previous studies have also demonstrated an association between frailty, cognition, and depression in older persons [43, 44].

Subgroup analysis of our variable of interest showed that negative transitions in individual components of the FI are associated with depressive symptomatology. Self-reported exhaustion was more significantly associated with depression in both men and women than other components of the FI. Some previous studies have revealed a strong correlation between vital exhaustion and depression [45, 46]. In addition, the impacts of the weakness of handgrip strength and social isolation on new-onset depressive symptoms have been investigated in previous research conducted in some countries [47, 48], including Korea [47, 49].

The etiology of the association between frailty and depression is not fully established. However, several possible explanatory mechanisms have been suggested. The findings of the above-mentioned studies support the concept of a uni- or bidirectional relationship between frailty and depression. However, interpretations of whether frailty and depression are causally related are limited owing to methodological weaknesses in the designs of the studies and the definitions and various measurement analyses of frailty status.

An alternative explanation for the considerable association between frailty and depression is that their indicators belong to overlapping domains of the same construct. Depressive symptoms are often included as some of several factors that constitute frailty measurement [50, 51]. Results of a previous confirmatory factor analysis of the indicators of depression and frailty suggested that these constructs capture distinct aspects of health, even though these aspects are highly related to each other [52]. The interdependence between frailty and depression may be explained by the impacts of their common causes, which exert similar effects on both of them. Therefore, frailty and depression may share a common susceptibility to the same factors, resulting in a significant association between them [53].

The current study has several limitations. First, all the data was self-reported and collected via survey, thus, we cannot exclude the risk of biased results. Second, the data of those who did not answer the essential covariate questions and those with cognitive impairment and depression at the baseline were excluded. We attempted to minimize the potential bias attributable to missing data by the employment of the imputation-based approach presented in the Supplementary materials, however, we cannot entirely eliminate the possible misestimation of the findings resulting in lower generalizability of the study findings. Third, biological risk factors that might significantly affect variables adjustment could be overlooked. Lastly, although the FI was developed and validated in the Korean population, the measure of frailty used in this study is not a universally used instrument. Furthermore, as this scale depends on self-reported estimation towards social and psychological aspects, personal or cultural differences may lead to information bias. Finally, the overlap of the exhaustion item with the CES-D-10 scale may also lead to a misestimation of found results. Further research using a broadly acceptable frailty measuring approach with higher validity and reliability measures are warranted.

Nonetheless, the strengths of our study include the relatively large sample size and longitudinal design, with results being representative of the Korean community-dwelling adult population over 60 years old. The panel data we employ allow us to temporally order our analysis to reduce the probability that associations between frailty and depression reflect its influence on the probability of becoming and remaining frail. Another strength is that the study provides an in-depth and broader view of frailty transition and related to its risk of depressive symptoms. Hence, exploring the dynamics of frailty status change over time on depression provides novel information compared to previous studies. The study provides longitudinal evidence to the growing body of literature that proposes that frailty and depression share common pathways and risk factors.

## Conclusions and implications

This study was conducted to assess the influence of frailty transitions on new-onset depressive symptoms using longitudinal, nationwide data of community-dwelling older adults in Korea. The findings of this study suggest that two-year frailty transitions are associated with new-onset depressive symptoms in older adults. Participants who transitioned into frailty or maintained a frailty status had a higher risk of depression than their non-frail counterparts. The results also demonstrated that exhaustion is a major component of the FI that leads to depression. Frail older adults who experience cognitive impairment showed stronger effects with depression. Early intervention and implementation of prevention strategies at physical, nutritional, and social levels are warranted to ameliorate frailty and depression in late life. Our study can contribute to the development of intervention strategies to better identify depression in later life of individuals who may be at greater risk due to their frailty conditions. Given that handgrip strength and social and psychological well-being can be measured at routine health check-ups, this study provides a substantial basis for policymakers to implement a frailty status screening through community-based healthcare programs for older people.

## Supplementary Information

**Additional file 1:** **Supplementary Table 1. **General characteristics of the study population (baseline 2008).

**Additional file 2:** **Supplementary Table 2. **Generalized linear model using the GEE with CES-D-10 score in 2008-2020 with employing imputation-based approach for missingdata.

**Additional file 3:** **Supplementary Table 3. **Generalized linear model using the GEE with CES-D-10 score in 2008-2020 without employing imputation-based approach formissing data.

**Additional file 4:** **Supplementary Table 4. **Generalized linear model using the GEE with CES-D-10 score in 2008-2020.

## Data Availability

The dataset supporting the conclusions of this article is available in the KLoSA repository, https://survey.keis.or.kr/klosa/klosa01.jsp.

## Abbreviations

OR: Odds ratio
CI: Confidence interval
GEE: Generalized estimating equation
KLoSA: Korean Longitudinal Study of Aging
MMSE: Mini-Mental State Examination
FI: Frailty instrument
CES-D-10: Center for Epidemiological Studies Depression 10 Scale
ADL: Limitations in activities of daily living
IADL: Limitations in Instrumental Activities of Daily Living
