# Supplementary material for: Frailty transition and depression among community-dwelling older adults: the Korean Longitudinal Study of Aging (2006–2020)
Source: BMC Geriatr. 2023 Mar 17;23:148. doi: 10.1186/s12877-022-03570-x (PMC10024357; doi:10.1186/s12877-022-03570-x)
Supplement: Supplementary file 1 — Additional file 1: Supplementary Table 1. General characteristics of the study population (baseline 2008). [file 12877_2022_3570_MOESM1_ESM.doc]

| **Supplementary Table 1. General characteristics of the study population (baseline 2008)** | | | | | | | | | | |
| --- | --- | --- | --- | --- | --- | --- | --- | --- | --- | --- |
| **Variables** | **Center of Epidemiologic Studies Depression Scale, 10-item version (CES-D-10)** | | | | | | | | | |
|  | **Men** | | | | | **Women** | | | | |
|  | **N** | **(%)** | **Median** | **IQR** | **P-value** | **N** | **(%)** | **Median** | **IQR** | **P-value** |
| **Total N=2 256** | **1,256** | **100.0** | **1.0** | **2.0** |  | **1000** | **100.0** | **2.0** | **2.0** |  |
| **Frailty status** |  |  |  |  | <.0001 |  |  |  |  | <.0001 |
| Non-frail → Non-frail | 1112 | 88.5 | 2.0 | 1.0 |  | 847 | 84.7 | 2.0 | 2.0 |  |
| Non-frail → Frail | 84 | 6.7 | 3.0 | 4.0 |  | 88 | 8.8 | 5.0 | 5.0 |  |
| Frail → Frail | 20 | 1.6 | 2.5 | 4.5 |  | 25 | 2.5 | 2.0 | 4.0 |  |
| Frail → Non-frail | 40 | 3.2 | 2.0 | 2.0 |  | 40 | 4.0 | 2.0 | 2.0 |  |
