# Supplementary material for: Frailty transition and depression among community-dwelling older adults: the Korean Longitudinal Study of Aging (2006–2020)
Source: BMC Geriatr. 2023 Mar 17;23:148. doi: 10.1186/s12877-022-03570-x (PMC10024357; doi:10.1186/s12877-022-03570-x)
Supplement: Supplementary file 3 — Additional file 3: Supplementary Table 3. Generalized linear model using the GEE with CES-D-10 score in 2008-2020 without employing imputation-based approach formissing data. [file 12877_2022_3570_MOESM3_ESM.doc]

| **Supplementary Table 3. Generalized linear model using the GEE with CES-D-10 score in 2008-2020 without employing imputation-based approach for missing data** | | | | | | | | | | |
| --- | --- | --- | --- | --- | --- | --- | --- | --- | --- | --- |
| **Variables** | **CES-D-10 score≥4** | | | | | | | | |  |
|  | **Men** | | | | | **Women** | | | | |
|  | **OR** | **95% CI** | | | ***p-value*** | **OR** | **95% CI** | | | ***p-value*** |
| **Including all participants starting from 45 years old without the exclusion of participants with cognitive impairment and depression at the baseline** | | | | | | | | | | |
| **Frailty status** |  |  |  |  |  |  |  |  |  |  |
| Non-frail → Non-frail | 1.00 |  |  |  |  | 1.00 |  |  |  |  |
| Non-frail → Frail | 1.30 | (1.25 | - | 1.35) | <.0001 | 1.32 | (1.28 | - | 1.36) | <.0001 |
| Frail → Frail | 1.34 | (1.26 | - | 1.42) | <.0001 | 1.48 | (1.42 | - | 1.55) | <.0001 |
| Frail → Non-frail | 1.03 | (1.00 | - | 1.07) | 0.0736 | 1.05 | (1.02 | - | 1.08) | 0.0010 |
| **Including all participants starting from 60 years old without the exclusion of participants with cognitive impairment and depression at the baseline** | | | | | | | | | | |
| **Frailty status** |  |  |  |  |  |  |  |  |  |  |
| Non-frail → Non-frail | 1.00 |  |  |  |  | 1.00 |  |  |  |  |
| Non-frail → Frail | 1.28 | (1.23 | - | 1.34) | <.0001 | 1.31 | (1.26 | - | 1.35) | <.0001 |
| Frail → Frail | 1.33 | (1.24 | - | 1.41) | <.0001 | 1.48 | (1.41 | - | 1.55) | <.0001 |
| Frail → Non-frail | 1.05 | (1.01 | - | 1.09) | 0.0131 | 1.04 | (1.01 | - | 1.07) | 0.0151 |
| **Including all participants starting from 60 years old with the exclusion of participants with cognitive impairment and depression at the baseline** | | | | | | | | | | |
| **Frailty status** |  |  |  |  |  |  |  |  |  |  |
| Non-frail → Non-frail | 1.00 |  |  |  |  | 1.00 |  |  |  |  |
| Non-frail → Frail | 1.26 | (1.21 | - | 1.32) | <.0001 | 1.34 | (1.28 | - | 1.40) | <.0001 |
| Frail → Frail | 1.29 | (1.21 | - | 1.38) | <.0001 | 1.51 | (1.41 | - | 1.62) | <.0001 |
| Frail → Non-frail | 1.04 | (1.00 | - | 1.08) | 0.0459 | 1.00 | (0.96 | - | 1.04) | 0.1464 |
| *All variables from the main analysis were included in the GEE model | | | | | |  |  |  |  |  |
