# Supplementary material for: Frailty transition and depression among community-dwelling older adults: the Korean Longitudinal Study of Aging (2006–2020)
Source: BMC Geriatr. 2023 Mar 17;23:148. doi: 10.1186/s12877-022-03570-x (PMC10024357; doi:10.1186/s12877-022-03570-x)
Supplement: Supplementary file 4 — Additional file 4: Supplementary Table 4. Generalized linear model using the GEE with CES-D-10 score in 2008-2020. [file 12877_2022_3570_MOESM4_ESM.doc]

| **Supplementary Table 4. Generalized linear model using the GEE with CES-D-10 score in 2008-2020** | | | | | | | | | | |
| --- | --- | --- | --- | --- | --- | --- | --- | --- | --- | --- |
| **Variables** | **CES-D-10 score≥4** | | | | | | | | |  |
|  | **Men** | | | | | **Women** | | | | |
|  | **OR** | **95% CI** | | | ***p-value*** | **OR** | **95% CI** | | | ***p-value*** |
| **Frailty instrument of 2 items: handgrip strength and social activity** | | | | | | | | | | |
| **Frailty status** |  |  |  |  |  |  |  |  |  |  |
| Non-frail → Non-frail | 1.00 |  |  |  |  | 1.00 |  |  |  |  |
| Non-frail → Frail | 1.06 | (1.01 | - | 1.11) | 0.0270 | 1.06 | (1.01 | - | 1.12) | 0.0111 |
| Frail → Frail | 1.11 | (1.01 | - | 1.21) | 0.0275 | 1.22 | (1.11 | - | 1.34) | <.0001 |
| Frail → Non-frail | 1.04 | (0.98 | - | 1.09) | 0.2178 | 1.06 | (1.00 | - | 1.13) | 0.0400 |
| *All variables from the main analysis were included in the GEE model | | | | | |  |  |  |  |  |
